# Advances in principles and technologies of non-mechanical blood pressure monitoring

**DOI:** 10.1038/s44325-025-00102-5

**Published:** 2026-02-27

**Authors:** Zixin Zheng, He Hao, Yiwen Huang, Rui Lyu, Huadong Ma, Yuqing Zhang, Jing Liu, Chunli Shao, Anfu Zhou

**Affiliations:** 1 Beijing University of Posts and Telecommunications, State Key Laboratory of Network and Switching Technology, Beijing, Beijing, China; 2 Fu Wai Hospital and Cardiovascular Institute, Department of Cardiology, Beijing, Beijing, China; 3 Chinese Academy of Medical Sciences & Peking Union Medical College, Beijing, Beijing, China; 4 Peking University People’s Hospital, Department of Hypertension, Beijing, Beijing, China; 5 State Key Laboratory of Vascular Homeostasis and Remodeling, Department of Cardiology and Institute of Vascular Medicine, Peking University Third Hospital, Beijing, Beijing, China

**Keywords:** Cardiology, Computational biology and bioinformatics, Engineering, Health care, Mathematics and computing

## Abstract

Intelligent blood pressure monitoring is transforming hypertension management. In this review, we introduce a novel classification based on measurement principles, categorizing devices into mechanical and non-mechanical methods. We offer fresh insights into the non-mechanical pipeline: advanced sensors, data preprocessing, modeling algorithms, and calibration strategies. We analyze clinical validation standards and limitations, highlighting the pressing need for updated frameworks tailored to next-generation cuffless systems *blood pressure, non-mechanical monitoring, deep learning*.

## Introduction

Hypertension *is a major global concern and a major risk factor* for cardiovascular diseases. According to the World Health Organization (WHO), an estimated 1.4 billion people aged 30–79 years were living with hypertension in 2024^1^. Hypertension notably elevates the risk of severe complications, including stroke, coronary artery disease, heart failure, and end-stage renal disease. However, nearly 46% are unaware of their condition, and over half are not receiving the treatment they need^2^. The alarming contrast between the high prevalence of hypertension and the low rate of awareness underscores a critical gap in global health management. A key step in addressing this challenge lies in ensuring accurate and accessible blood pressure (BP) measurement methods^3^.

**Fig. 1:**
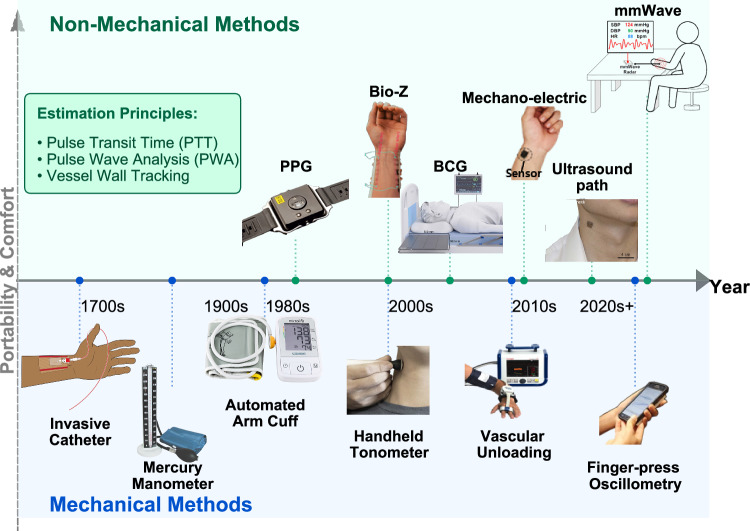
Development history of BP measurement devices. Key milestones in the evolution of blood pressure monitoring. The timeline highlights representative devices and time points. Images are adapted with permission from Springer Nature^50^^,^^65^^,^^175^^,^^177^^,^^178^, AAAS^179^^,^^180^, and ACM^14^. The Invasive Catheter, Mercury Manometer, and Automated Arm Cuff images are courtesy of Red Roan, Michael V Hayes, and Jacek Halicki, respectively, used under CC BY-SA licenses.

As shown in the timeline in Fig. 1, the history of BP monitoring reflects a continuous evolution driven by the need of better accessibility and comfort. Invasive BP measurement, typically performed at the radial or femoral artery, was first performed in the 18th century and remains the clinical reference method due to its accuracy^4^. However, it is confined to surgical and critical care settings due to the need for intra-arterial cannulation. To enable routine monitoring, the auscultatory method introduced in 1905 by detecting Korotkoff sounds through a compressed brachial artery. Although it remains the gold standard for non-invasive measurement, it requires trained personnel and is prone to subjective interpretation. The oscillometric method developed in 1976 automated this process by analyzing the pressure oscillation envelope at the upper arm or wrist to estimate BP^5^. While these cuff-based methods are invaluable, they share critical drawbacks. First, they require high-pressure cuff inflation, which can cause discomfort and disturbance, limiting the use for frequent monitoring^6^. Second, they can only provide readings under static conditions, making them unsuitable to capture dynamic BP fluctuations induced by daily physical or psychological activity^7^.

In the last few years, BP measurement technology has been accelerating rapidly, driven by the advance in consumer electronics and new sensors. In particular, BP measurement has been integrated into different kinds of wearable devices, such as patches^8^, smart watches^9^, rings^10^, in-ear devices^11^, and neckbands^12^ so as to enable ubiquitous BP tracking. Beyond wearable devices, there is a growing trend towards fully contact-less solutions. Recent work has demonstrated the feasibility of estimating BP by contactless sensors, such as remote photoplethysmography (rPPG)^13^ and millimeter-wave (mmWave) sensors^14,15^. To navigate the wide range of BP measurement methods, this paper presents a systematic review from the new perspective of measurement principles, with a particular focus on the rapidly emerging approaches for convenient and continuous BP monitoring. Unlike existing review works, our work differs in two key aspects:

### New perspective on categorizing existing works and the consequent new insights

Previous reviews typically classified BP technologies by device form factors—such as wearable or cuff-less designs—which reflect usage scenarios^16–21^. In contrast, we propose a more fundamental classification based on whether the final BP values are obtained through direct pressure measurement or indirect estimation from physiological surrogates. This divides into *mechanical* and *non-mechanical*. Mechanical methods rely on force transduction through physical application (occlusion or applanation). The measurement directly correlates with physical pressure, and the output is expressed in the standard unit of mmHg. While non-mechanical methods estimate BP from measured physiological signals (e.g., pulse waveform, vascular radius). These signals serve as surrogates, and the final BP value is derived via a computational model. This categorization offers new insights, as the two types differ significantly in sensing, modeling, and signal interpretation. We focus on non-mechanical methods and provide an in-depth analysis of their full processing pipeline: signal acquisition, preprocessing, modeling, and calibration.

### Analysis on clinical validation

Despite the promising potential of non-mechanical technologies, significant challenges remain in translating current technologies into clinical applications. To bridge this gap, we conduct a comprehensive investigation into clinical validation, an often overlooked aspect in previous reviews. We summarize international validation standards, outlining their limitations and applicability to underscore the need for revised frameworks tailored to next-generation BP monitoring systems. We also examine both academic prototypes and commercial devices that have undergone clinical validation, highlighting the extent of their compliance with criteria, and common shortcomings.

## Measurement Principle

Based on the fundamental principle of BP derivation, the emerging intelligent BP monitoring has evolved into two conceptual categories: Mechanical Methods and Non-mechanical Methods, as visually summarized in Fig. 1.

### Mechanical Methods

This category, depicted by the blue arrow in Fig. 1, encompasses representative techniques whose unifying principle is the active application of external mechanical pressure to the artery. These methods rely on force transduction, where the measurement directly correlates with the physical pressure. Historically, this category includes Invasive catheter^22^, classic cuff devices, such as the Mercury Manometer^23^, and Automated Arm Cuff devices^24^. More recently, this classification extends to continuous methods based on applanation tonometry^25^ and the vascular unloading principle ^26^^,^^27^. It is worth noting that the necessity of implementing external pressure defines the classification. Although methods like Vascular Unloading and emerging Finger-press Oscillometry^28^ utilize advanced sensors (like PPG) for system control or oscillation detection, their core function requires the active application of external counter-pressure for measurement.

### Non-mechanical Methods

In contrast, the Non-mechanical category, shown by the green arrow, infers BP values by measuring related physiological parameters that serve as surrogates. These signals are then processed and modeled to derive the final BP value. They basically rely on the principles of the following three methods:

#### Vessel Wall Tracking

During the cardiac cycle, heart contraction leads to the increased BP, resulting in the expansion of blood vessels, while relaxation leads to a decrease in blood flow and subsequent vessel contraction. Thus, by tracking the changes in the vessel wall diameter and transforming them into the corresponding BP waveform, BP values can be obtained^29^. This relationship is locally dependent and is often applied to skin regions capable of clearly detecting the target artery’s pulse, such as the forearm, brachium, neck, chest, abdomen^30^. The mathematical transformation can be represented by:

1 $$p(t)={p}_{D}\cdot {e}^{(\alpha \frac{A(t)}{{A}_{D}}-1)}$$

where $p(t)$ and $A(t)$ represent the BP values and arterial cross-section at time $t$ at the measured site, respectively, while ${p}_{D}$ is DBP and ${A}_{D}$ is the diastolic arterial cross-section at the measured site. The coefficient $\alpha$ represents the vessel rigidity, and can be calculated as:

2 $$\alpha =\frac{{A}_{D}\cdot (ln{p}_{S}-ln{p}_{D})}{{A}_{S}-{A}_{D}}$$

where ${p}_{S}$ is SBP and ${A}_{S}$ is systolic arterial cross-section. $A(t)$ can then be calculated as:

3 $$A(t)=\frac{\pi {d}^{2}(t)}{4}$$

where $d(t)$ is the radius waveform of the measured artery.

#### Pulse Wave Velocity

Pulse Wave Velocity (PWV) is defined as the velocity of pulse wave propagation in the artery. It serves as an important clinical indicator for assessing vascular function and predicting the risk of cardiovascular events^31,32^. Extensive experiments have demonstrated that higher PWV is associated with a greater risk of hypertension^33^. Besides, the variation of PWV can be utilized to correlate the absolute BP change individually^34^. PWV is typically represented by the Moens-Korteweg (MK) equation^35^:

4 $${PWV}=\sqrt{\frac{{Eh}}{\rho {d}_{D}}}$$

where $E$ is the modulus of elasticity, $h$ is the thickness of arterial wall, $\rho$ is the blood density and ${d}_{D}$ is the diameter of the artery at the end of diastole. As a more accessible approach to BP estimation, Pulse Transit Time (PTT) refers to the time it takes for the pulse wave to travel through a segment of the arterial tree, typically measured as the delay between the foot points of pulse waveforms obtained at two distinct anatomical sites^36^. A related and often more practical metric is Pulse Arrival Time (PAT)^37^. PAT is defined as the interval from the cardiac electrical activation (*e.g*. ECG R-wave on the chest) to the arrival of the pulse at a peripheral artery, such as the finger^32^. Additional variants also can characterize pulse wave timing within the vasculature. For example, Reflection Wave Transit Time (RWTT) measures the interval or amplitude difference between the forward and reflected pulse waves at the same anatomical site^38,39^.

#### Pulse Wave Analysis

With the development of machine learning and non-mechanical devices, Pulse Wave Analysis (PWA) has become a focal area for estimating BP values by analyzing the shape and characteristics of the pulse waveform. Analysis involves methods ranging from extracting explicit temporal and morphological features^40,41^ to leveraging deep learning for end-to-end signal analysis^42^. While PWA differs from the above methods in that it relies less on a single explicit bio-mechanical equation, extensive data-driven evidence demonstrates that waveform morphology inherently and robustly encodes critical hemodynamic status, including cardiovascular characteristics, such as arterial elasticity and stiffness^43^.

## Data Acquisition

**Fig. 2:**
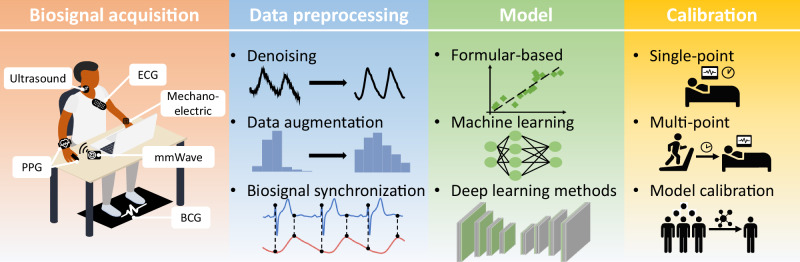
Workflow of intelligent non-mechanical blood pressure monitoring. The process involves four critical stages: sensors to capture biosignals, preprocessing to enhance signal quality, estimation modeling to derive BP values, and calibration to ensure accuracy across individuals and conditions.

As illustrated in Fig. 2, the typical process flow for non-mechanical BP estimation encompasses four key phases: (*i*) acquiring biosignals using sensors, (*ii*) preprocessing the biosignals, (*iii*) modeling the relationship between the biosignals and BP, and (*iv*) calibration. Each of these components will be addressed in detail. The first step is to collect effective BP-related biosignals that reflect cardiovascular activity. We will introduce the existing novel non-mechanical sensors and the principle of how they obtain biosignals in Section 3.1. Then, we introduce the available large-scale datasets and their characteristics in Section 3.2.

### Advances in Biosignal Acquisition

**Fig. 3:**
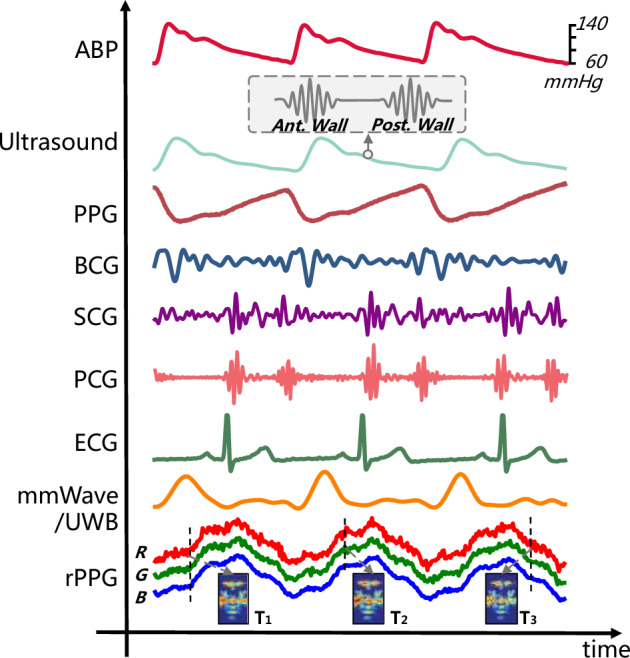
Illustration of the simultaneous acquisition and alignment of various non-mechanical biosignals with the invasive arterial blood pressure reference waveform (ABP). rPPG images adapted from^13^ (Wolters Kluwer Health).

With the advancement of technology, a growing diversity of techniques for obtaining biosignals has emerged. These methods can be categorized into two types based on the requirement of physical contact with the skin: Contact-Based methods require direct physical coupling between the sensor and the skin, generally offering high signal fidelity but potentially compromising long-term comfort or adherence. Conversely, contactless methods operate remotely, significantly enhancing user comfort and enabling passive, long-term monitoring. However, these methods often require complex signal processing to mitigate ambient noise and interference. The following sections detail the acquisition principles and representative examples for each type of monitoring. The vast array of data modalities relevant to these two categories is visually summarized in Fig. 3. This figure provides an overview of the key biosignals used for non-mechanical BP estimation, demonstrating the distinct morphology and temporal synchronization of signals obtained via both contact-based and contactless acquisition methods^44–49^.

#### Contact-based Devices

*Ultrasound* operates by generating high-frequency sound waves that can penetrate the body and reflect off internal structures. As a widely used diagnostic tool in medical imaging, ultrasound can also be applied to assess BP values by measuring the dynamic changes in arterial diameter. To achieve this, the ultrasound probe or patch is placed over specific arterial sites, typically the carotid artery (CA), brachial artery (BA), and radial artery (RA). High-frequency sound waves are then emitted and travel through tissues, reflected back to the transducer upon encountering interfaces including the vessel walls. By continuously recording the time taken for the echo to return, the device can locate the dynamic change of anterior and posterior vessel walls and estimate BP values according to vessel wall tracking method as mentioned in Section 2.2.1. The recent emergence of wearable ultrasound patches^50,51^ adopts ultra-thin flexible circuits and stretchable sensor arrays, enabling continuous and non-invasive monitoring. However, the cost of single use for such wearable solutions remains relatively high.

*Photoplethysmography (PPG)* is one of the most extensively explored technologies for BP measurement^52,53^. It uses light to probe blood volume changes beneath the skin of the ear canal, fingers, and wrist^54,55^. As light passes through tissue, its absorption varies with pulsatile blood flow. During systole, increased arterial blood volume leads to greater absorption; during diastole, absorption decreases accordingly^56^. By analyzing these signal fluctuations, signals regarding cardiovascular health and hemodynamic parameters can be extracted. However, variations in skin tone and thickness can affect the quality of PPG signals, as darker skin tones and thicker skin layers tend to absorb more light and reduce signal strength^57^. The pressure applied to PPG sensor can also impact the amplitude and shifts of signals^58^. A potential solution is to integrate a sensor capable of measuring the applied force, which allows for standardizing PPG measurements across different users and conditions^59^.

*Seismocardiography (SCG) and ballistocardiography (BCG)* detect the subtle mechanical vibrations generated by the heart’s activity, including contractions and blood flow within each heartbeat. SCG typically uses an accelerometer placed on the chest to capture the mechanical vibrations from the chest wall due to heart activity^60,61^. Devices for BCG measurement typically employ highly sensitive sensors, such as electromechanical^62^, accelerometric^63^, piezoelectric^64^, fiber-optic^65^^,66^ and hydraulic sensors^67^. These BCG sensors are positioned unobtrusively beneath the user on surfaces, such as mattresses, chairs, or insoles, capturing pressure changes in the body at the wrist and feet, as well as in sitting and lying postures, without requiring direct contact with user. Due to its high sensitivity to external movements and vibrations, SCG and BCG are prone to noise and artifacts, which can interfere with the clarity of signal^68,69^.

*Phonocardiogram (PCG)* measures the acoustic manifestations of the cardiac cycle, *i.e*. the heart sounds ($\mathrm{S}1$ and $\mathrm{S}2$), which are generated by the mechanical action of valve closure and blood flow turbulence^70^. PCG is acquired using highly sensitive microphones or acoustic sensors placed on the chest or neck. It can provide precise timing markers (e.g., $\mathrm{S}1$) for the accurate decomposition of $\mathrm{PAT}$ when combined with other sensors^71^. In addition, the $\mathrm{PCG}$ waveform itself is utilized in end-to-end deep learning models for BP prediction^72^. However, heart sound signals are extremely weak (usually between 20-200 Hz) and are easily drowned out by external sounds.

*Deformable wearable mechanoelectric sensors* convert mechanical stimuli from arterial deformation or vibration into electrical signals using various transduction mechanisms. They are typically placed on the radial artery or brachial artery to detect pulse waves. Common types include piezoelectric (stress-induced charge generation)^73,74^, triboelectric (contact-induced charge transfer)^75^, piezoresistive (pressure-induced resistance change)^76,77^, and capacitive (deformation-driven capacitance change) sensors^78^. While these patch-based sensors offer high sensitivity, ensuring stable, conformal contact on moving skin and long-term durability remains a key research challenge.

*Bioimpedance (Bio-Z)* is typically performed using a four-electrode configuration to enhance measurement accuracy. Two outer electrodes inject a small alternating current, while two inner electrodes measure the resulting voltage to derive the impedance value. When these four electrodes are aligned with the objective artery, they measure the underlying tissue bioimpedance, which typically ranges from $10-100\Omega$. The fundamental principle is that the change in impedance is inversely related to the pulsatile change in blood volume, generating a pulse bioimpedance waveform that reflects the pressure pulse wave^79,80^.

*Electrocardiogram (ECG)* captures heart activity by detecting electrical impulses, serving as a highly precise timing reference in cardiovascular measurements. Electrodes are accurately placed on the skin of the chest and limbs to measure the potential difference. The distinct $\mathrm{R}$-wave in the $\mathrm{ECG}$, corresponding to ventricular depolarization, is utilized as the reliable fiducial point for starting time-interval measurements, such as PAT^81,82^. In wearable applications, simplified single-lead configurations, often taken across the chest or wrist-to-wrist, are typically employed in conjunction with other sensors to calculate $\mathrm{PAT}$.

#### Contactless Devices

*RF signals*, including Ultra-Wideband (UWB), and millimeter-wave (mmWave) radar, have gained popularity in BP estimation. The radar system emits electromagnetic waves that can penetrate clothing, with some of the energy being absorbed by the body and a portion reflected back to the receiving antenna^83^. By analyzing the phase changes and signal strength of the reflected signals, the system precisely tracks the minor displacement of the chest wall or the arterial skin surface. UWB signals offer high temporal resolution and have been integrated into existing smart devices^84,85^. mmWave radar (typically in the 60 GHz, or 77 GHz bands in BP estimation) provides even finer spatial and temporal resolution thanks to its short wavelength and wide bandwidth^14,86,87^. These RF-based approaches enable unobtrusive BP monitoring without direct skin contact, though challenges remain in susceptibility to environmental interference and precise sensor positioning.

*Remote photoplethysmography (rPPG)* operates on principles similar to contact-based PPG, which employs an RGB camera and a light source to capture color video of the skin region, typically the face or palm. The system identifies regions of interest within each frame and extracts raw signals from different color channels. By analyzing the fluctuations of signals over time, rPPG can derive physiological parameters, such as heart rate and BP values^88,89^. However, challenges remain in ensuring consistent performance under varying environmental conditions and skin tones, which can affect signal quality and accuracy^90^.

### Public Dataset

In addition to building custom systems using the aforementioned sensing methods, there are also publicly available biosignal-BP datasets that can facilitate research. Table 1 summarizes commonly used datasets that are invaluable for developing reliable BP estimation models. In experimental and testing processes, accounting for factors such as sample size, demographic diversity, and cardiovascular disease risk among subjects can significantly advance the applicability of intelligent BP measurement systems to clinical settings.

**Table 1:** Subject size and characteristics of public datasets

Dataset	No. of subjects	GT Device	Modalities	Demographic	Note
MIMIC Ⅳ dataset^163^	364,627	cNIBP, cIBP	ECG, PPG, CVP, tonometer	Age, height, weight, gender, EMR	Data all comes from all patients admitted to the intensive care unit of Beth Israel Deaconess Medical Center between 2008 and 2019.
MIMIC Ⅲ dataset^164^	40,000	cNIBP, cIBP	ECG, PPG, CVP, tonometer	Age, height, weight, gender, EMR	Data all comes from all patients admitted to the intensive care unit of Beth Israel Deaconess Medical Center between 2001 and 2012.
MIMIC Ⅱ dataset^165^	25,328	cNIBP, cIBP	ECG, PPG, CVP, tonometer	Age, height, weight, gender, EMR	Data all comes from all adult patients admitted to the ICU at Beth Israel Deaconess Medical Center in Boston between 2001 and 2007.
VitalDB^166^	6388	cNIBP	ECG, PPG, tonometer	Age, height, weight, gender, EMR	Data comes from surgical patients who undergo non-cardiac surgery, and provides over 60 clinical information.
CAS-BP^144^	1272	Auscultatory	ECG, PPG, tonometer	Age, height, weight, gender, hypertension history	Each subject underwent multiple measurements at D, D + 7, D + 14, and D + 21.
Aurora-BP^167^	1125	Auscultatory, oscillometric	ECG, PPG, tonometer	Age, height, weight, gender, CVD history	Dataset includes data during nighttime sleep, raising arms, running test, etc.
Automatic Aging^168,169^	1121	cNIBP	ECG	Age, gender	Dataset focuses on systematic analysis of the impact of healthy aging on the cardiovascular system.
PPG-BP^170^	219	Oscillometric	PPG	Age, height, weight, gender, CVD history	Dataset covers diseases including hypertension, diabetes, cerebral infarction, and insufficient brain blood supply.
Blumio^171^	115	cNIBP	mmWave, PPG, tonometer	Age, BMI, gender, pregnancy status, medicated for hypertension	Dataset also provides specially processed mmWave phase signals.
MSMP^172^	103	Oscillometric	rPPG, near-infrared (NIR)	Age, BMI, gender, ethnicity, smoking status, caffeine consumption, makeup usage	Dataset captures full-body video synchronized with multi-site contact PPG and incorporates robust hemodynamic protocols, such as guided respiration.

(Note: *GT* ground truth, *cNIBP* continuous non-invasive blood pressure, *cIBP* continuous invasive blood pressure, *ECG* electrocardiogram, *PPG* photoplethysmogram, *CVP* central venous pressure, *EMR* electronic medical records, *CVD* cardiovascular disease, *BMI* body mass index, *NIR* near-infrared).

## Data Preprocessing

After obtaining biosignals, conducting data preprocessing is crucial for enhancing the reliability and robustness of BP measurement systems. In section 4.1, we introduce methods for denoising to remove environmental and physiological noise. Section 4.2 covers data augmentation techniques used to expand and diversify training datasets for more robust model performance, while Section 4.3 focuses on aligning multimodal data streams accurately across devices.

### Denoising Technique

Physiological signals are often contaminated with various types of noise, including inherent sensor properties, such as power line interference, baseline wander, motion artifacts and ambient noise. These noises can obscure critical features of the signal, making accurate analysis and interpretation challenging. To prevent outliers from impacting subsequent processing, outlier detection and segment rejection are typically the first step to clean severely affected parts of the signal. Manual checks are used to exclude irregular and distorted segments, while the autocorrelation filters can discard corrupted segments^85^. The most common approach for further signal refinement involves traditional filtering techniques, which effectively suppress noise and enhance signal quality^91^. Tailored noise suppression strategies have also been developed for different sensing modalities. For example, for PPG, adaptive light intensity control is often used to balance baseline levels while preserving pulsatile components^92^. In mmWave sensing, multi-antenna phase compensation techniques can enhance pulse signal coherence^93^. Recent advances in deep learning have introduced data-driven denoising methods that learn to separate physiological signals from noise based on large training datasets^94,95^.

### Data Augmentation

Due to the challenges in collecting extensive high-quality BP-signal data, data augmentation through variations of existing data is essential. One approach involves applying transformations to current data, ranging from traditional methods (such as flipping and cropping) to deep learning methods^96^. Another approach involves gathering diverse data details from a smaller subset and generalizing these variations across the dataset. By collecting signals at varying intensities, positions, and angles and utilize the different waveform patterns to unify the measurement in different situations^61,97^. In addition, BP datasets often suffer from imbalances. Certain BP ranges or personal information can restrict the output of model to the distribution of the training data^98^. Generating synthetic waveform data using deep learning can create more diverse and balanced dataset, enabling the model to learn a broader range of patterns and improving its robustness to real-world variations^99,100^.

### Synchronization Between Devices

In BP estimation works, multiple devices are often used to capture physiological signals. However, the temporal misalignment of data could lead to unreliability of the estimation algorithms, especially for the PTT-based methods since it is extremely short, ranging from only tens to hundreds of milliseconds ($\mathrm{ms}$)^101^. Hence, accurate synchronization of multiple devices is crucial in BP estimation. A common approach involves generating an external stimulus that can be detected simultaneously by all sensors, enabling precise alignment and calibration of timing. For instance, interrupt signals, physical taps, speaker-generated beeps, or fixed-length data frames are used as stimuli to ensure synchronization across different sensors^81^.

## Estimation Model

This section provides a comprehensive review of various models used for BP estimation, categorized into formula-based, machine learning, and deep learning methods.

### Formula-based methods

Back in 1976, the theoretical foundation of pulse wave velocity (PWV) dependence on arterial pressure was established, with experimental evidence supporting the linear relationship between PWV and BP^102^. Based on the Moens-Korteweg (M-K) equation and vessel elasticity theory^103^, logarithmic and exponential dependencies were derived under the assumption of constant vessel thickness and radius^104^. To enhance individual accuracy, modeling has incorporated $\mathrm{PTT}$ alongside other physiological parameters, such as Heart Rate ($\mathrm{HR}$) and morphological features^105^, providing the basis for the subsequent PTT-BP estimation algorithm.

### Machine learning-based methods

Machine learning methods can offer greater flexibility in capturing the nonlinear relationships between pulse waveform features and BP. Traditional machine learning methods include decision trees (DT)^106^, random forest regression (RFR)^107^, adaptive boosting(AdaBoost)^108^, and K-nearest neighbors (KNN)^109^. Nisio et al.^110^ extracts 195 features from PPG pulse waveforms and evaluates the performance of various machine learning models. Among them, gaussian process regression (GPR) can capture complex nonlinear relationships and XGBoost excels in robust gradient boosting. Experiments indicate that their combination achieves the optimal trade-off between training time and accuracy.

Machine learning methods typically require careful feature engineering, which can introduce inaccuracies due to variations in signal quality and the absence of key features. Ideally, a pulse waveform contains the systolic peak, reflected wave, and dicrotic notch in a sequential pattern^111^. However, in risky groups, such as elderly individuals or hypertensive patients, the reflected wave may shift or even precede the systolic peak^112^.

### Deep learning-based methods

Advanced deep learning methods utilize multi-layer neural networks to autonomously learn from large-scale datasets. This section discusses commonly used deep learning models in BP estimation algorithms, with representative studies, experimental scales, and results summarized in Table 2. Convolutional Neural Networks (CNN), originally designed for image processing, is highly effective in extracting hierarchical features from the pulse waveform. This process effectively replaces manual waveform feature extraction by finding optimal local feature maps across the pulse waveform^113–115^. Recurrent Neural Network (RNN) incorporates feedback connections to model temporal dependencies in sequential data^116^. Long Short-Term Memory (LSTM) network and the Gated Recurrent Unit (GRU) significantly improve upon RNN by using sophisticated gating mechanisms. They are particularly effective at modeling the long-term temporal dynamics and sequence-to-sequence relationships inherent in continuous pulse waveforms^117–119^. More advanced DL frameworks utilize specialized structures to refine sequential processing and fusion. The Encoder-Decoder framework processes the input signal sequence into a compressed latent representation, which serves as a compact summary of the underlying hemodynamic state, and then reconstructs the desired output or feature map^120–122^. The Attention mechanism is a crucial breakthrough. By allowing the decoder to selectively and dynamically focus on critical, BP-relevant feature points within the input sequence, it significantly enhances predictive performance^123–125^. Beyond traditional predictive modeling, Physics-Informed Neural Network (PINN) integrates theoretical rigor by incorporating known physical laws, such as the $\mathrm{Moens}-\mathrm{Korteweg}$ equation, directly into the network’s loss function. By enforcing adherence to these physical constraints during training, $\mathrm{PINN}$ ensures that the resulting $\mathrm{BP}$ predictions are physically plausible, thereby enhancing the generalization ability and interpretability of the model^126,127^.

Although a single physiological modality provides valuable advancement, it inherently provides only a partial view of the complex cardiovascular system, and multimodal fusion has become increasingly popular. As Fig. 3 shows, each modality captures a distinct aspect of the cardiac cycle. For example, $\mathrm{ECG}$ provides precise electrical timing, $\mathrm{PPG}$ reflects peripheral volumetric changes, while $\mathrm{mmWave}$ quantifies vibration momentum. By leveraging the complementary physiological information from different sensor modalities, fusion strategies mitigate individual sensor limitations. In addition to improving accuracy, this enhances resilience by compensating for modality-specific biases (such as $\mathrm{PPG}$’s sensitivity to skin tone), thereby achieving a more robust representation of the underlying hemodynamic state^128–130^.

**Table 2:** Summary of research on deep learning-based methods

Ref.	Model	Cali.	Modality	Input	Dataset source	#Sub.	Performance (ME ± STD) [mmHg]
^13^	FNN	+	rPPG	Features	Lab dataset & Physical center	1328	SBP: 0.39 ± 7.30 DBP: -0.20 ± 5.00
^173^	CNN	-	ECG, PPG	Waveform	MIMIC II^165^	379	SBP: 0.13 ± 7.54 DBP: -1.23 ± 12.80
^174^	ResLSTM	-	ECG	Waveform	MIMIC III^164^	428	SBP: -0.11 ± 9.99 DBP: -0.03 ± 6.36
^121^	Transformer	-	PPG	Waveform	CAS-BP dataset^144^	1272	SBP: 0.70 ± 8.30 DBP: -0.90 ± 6.50
^123^	Attention	-	mmWave	Waveform	Hospital & Lab dataset	1012	SBP: -1.57 ± 9.77 DBP: -0.34 ± 7.93
^127^	PINN	+	bioimpedance	Waveform	Bio-Z datasets^108,175,176^	15	SBP: 1.30 ± 7.60 DBP: 0.60 ± 6.40
		-					SBP: 0.20 ± 12.10 DBP: 0.60 ± 8.70

(Note: *SBP* systolic blood pressure, *DBP* diastolic blood pressure, *ME* mean error, *STD* standard deviation, *FNN* feedforward neural network, *CNN* convolutional neural network, *ResLSTM* residual long short-term memory, *PINN* physics-informed neural network, *rPPG* remote photoplethysmography, *ECG* electrocardiogram, *mmWave* millimeter wave. In the Calibration (Cali.) column, ‘+‘ indicates calibration is required, while ‘-‘ indicates a calibration-free method).

## Calibration

Unlike traditional oscilloscope devices that directly provide absolute BP values, cuff-less BP monitoring is particularly sensitive to individual physiological variations, such as posture, physical activity, emotional state, and long-term cardiovascular changes, as well as to environmental factors and sensor variability. Therefore, calibration is essential to assist in mapping the recorded signals to actual BP values. The calibration strategies can generally be divided into three categories:

### Single-point calibration

These methods involve repeatedly measuring BP under the same physiological and environmental conditions and selecting part of these measurements from the same subject as the calibration standard and the rest serveing as verification. For instance, commercial solutions like Samsung Health Monitor^131^ and Somnotouch-NIBP^132^ mandate a rigorous calibration process before use. This process requires users to perform initial measurements against a medical-grade arm cuff (typically involving two to three comparison readings in a row) and necessitates periodic re-calibration (e.g., every 28 days for $\mathrm{Samsung}$) to mitigate calibration drift. These methods may struggle to accurately track fluctuations in BP changes under varying conditions. Hence, they require frequent re-calibration to maintain accuracy, which poses challenges in ensuring reliability during long-term monitoring^133^.

### Multi-point calibration

These methods introduce interventions that alter the environment or physiological state, allowing for a more comprehensive calibration by fitting multiple data points collected in different states. Huang et al.^134^ utilized a multi-point approach in laboratory settings, where the training data included signals collected during induced physiological changes (e.g., ice-water stimulation) to ensure a wide range of $\mathrm{BP}$ fluctuations is captured. For their long-term monitoring or $\mathrm{ICU}$ scenarios, this calibration is typically achieved by training the model on data collected during one distinct time segment and validating it on data from a later time segment, ensuring the model can adapt to temporal drift and maintain accuracy across different physiological states. Colburn et al. ^135^ presented a novel approach that leverages the hydrostatic pressure effect by adjusting the arm to different heights relative to heart level to induce pressure perturbations, thereby generating optimal PTT predictions using personalized calibration.

### Model calibration

This approach leverages a pre-trained model from a large dataset and adapts it to the target individual’s data. The generalization capabilities enable it to capture common physiological patterns across a population. Nguyen et al.^136^ proposed a three-stage model specifically designed for BP prediction in pregnant women. It involves a baseline model trained from the general population, fine-tuning it for the pregnant population, and further personalizing the model to enhance accuracy for individual subjects. Fan et al.^137^ designed a few-shot transfer learning approach and achieved MAE of 6.68 mmHg for SBP and 3.91 mmHg for DBP with only ten pairs of personal data samples in cold pressor and HIIT tests. Hu et al.^138^ propose a Cross-Modality Knowledge Transfer (CMKT) strategy that leverages a teacher model pre-trained on the MIMIC dataset (ECG/PPG) to calibrate a millimeter-wave student network, embedding generalized cardiac knowledge. To further address individual physiological heterogeneity, they employ a subject-specific personalization scheme that fine-tunes the generalized model using approximately 20 minutes of target data, significantly mitigating distribution shifts and reducing estimation errors.

However, there remain significant challenges in the practical application of calibration. Firstly, only when the reference values are accurate, can the predicted BP be reliable. Yet, home-use wrist and arm-based oscillometric devices are often prone to overestimation, and their accuracy can degrade further over time if not regularly re-calibrated^139^. Mehta et al.^140^ pointed out that several studies have employed short time-scale data for BP estimation, where the actual values tend to remain relatively stable. As a result, even if a model predicts constant values, it can still achieve deceptively high accuracy, giving the false impression of model effectiveness. Additionally, the accuracy levels of using discrete BP data points as reference values and continuous BP waveforms are different^141^, but using continuous waveforms is impractical in typical home-use monitoring devices. Secondly, there is limited systematica research conducted on the available frequency of calibration, and yet no standardized guidelines exist for determining how often calibration should be performed^142^. Han et al.^143^ investigated the accuracy of user-driven re-calibration, revealing a pre-post calibration absolute $\mathrm{SBP}$ difference of $6.8\pm 5.6$ mmHg (with a range up to $33.8\mathrm{mmHg}$). Crucially, this significant drift was not due to intrinsic device instability (found to be $\approx 0.02\mathrm{mmHg}/\mathrm{day}$), but was primarily influenced by changes in the participant’s average physiological $\mathrm{SBP}$ level prior to re-calibration. The 2023 ESH consensus highlights that the lack of accuracy in verification reinforces the necessity for frequent calibration. Fundamentally, these cuffless devices track changes relative to a calibration point or attempt to predict BP using demographic and machine learning techniques rather than measure BP. We advocate for more research to validate the effectiveness of methods through long-term monitoring application, specifically investigating the impact of calibration frequency and methods.

## Validation

Clinical validation ensures that the device performs reliably under practical conditions, accounting for physiological variability, environmental noise, and diverse patient populations—factors often absent in controlled lab experiments. However, most research remains in the laboratory validation stage to date, with only a few studies having conducted clinical validation of their devices to demonstrate their accuracy in specific clinical environments. Among them, studies ^144–149^ have undergone clinical validation and reported BP measurement results, though they did not align with international standards. Research and commercial devices including FreeScan^150^, LiveOne^151^, Lifelight^152^, CardioWatch^153^ have assessed accuracy based on the AAMI/ESH/ISO universal standard^154^. However, some studies have only met the error requirements, without fulling other essential criteria, such as including a minimum of 85 subjects or ensuring a sufficient representation of cases with abnormal BP values.

Recognizing the unique characteristics of cuffless devices in measurement principles, sensing modalities, functionalities, and calibration^154^, emphasizes the need for distinct validation criteria. The IEEE has released the standard protocol IEEE 1708-2014^155^ and it has been adopted in various studies for validation^65^. The most comprehensive recent effort comes from the European Society of Hypertension ($\mathrm{ESH}$) Working Group (2023)^156^. The $\mathrm{ESH}$ explicitly recommends six distinct validation tests for cuffless $\mathrm{BP}$ devices, moving validation beyond simple absolute accuracy: These tests include the static test (absolute $\mathrm{BP}$ accuracy), device position test (hydrostatic pressure effect robustness), treatment test ($\mathrm{BP}$ decrease accuracy), awake/asleep test ($\mathrm{BP}$ change accuracy), exercise test ($\mathrm{BP}$ increase accuracy), and the re-calibration test (stability over time). Successful completion of all required tests, depending on the device type, is necessary for clinical recommendation. Despite these significant advances, a universally accepted clinical protocol for all intelligent $\mathrm{BP}$ monitoring devices has not yet been achieved^8^.

## Future Direction

Despite significant progress in laboratory conditions, the clinical adoption of cuffless BP measurement has remained cautious due to the lack of full validation of its reliability. Improving technical methods, enhancing real-world accessibility, refining evaluation standards, and establishing clinical relevance are key directions to build clinical trust in the work of new researchers and ensure its application in mHealth.

- Integration with Large-Scale Foundation Models for Diagnostics. Existing work has already demonstrated the success trail of large models in complex medical tasks^157–159^. For $\mathrm{non}-\text{mechanical BP monitoring}$, we envision leveraging the capacity of Large Language Models (LLM) to integrate structured biosignals with rich clinical narratives and patient history. By processing these heterogeneous data types concurrently, the $\mathrm{LLM}$ could establish a context-aware unified representation of the patient’s hemodynamic state. This approach promises truly personalized, context-aware $\mathrm{BP}$ prediction, thus significantly enhancing the robustness and clinical utility of cuffless devices.
- Enhancing usability in diverse daily scenarios. Despite the portability of wearable devices, there remains room for improving 24 h comfort and achieving truly imperceptible designs. For contactless systems, future development should aim to reduce sensitivity to body posture and alignment without requiring active user adjustment. In addition, charging frequency for sensors requiring high sampling rates or continuous wireless transmission remains a critical limitation.
- Exploring new sensing modalities. New sensing technologies hold significant potential for improving the quality of pulse detection and BP measurement. For example, terahertz (THz) technology^160^, with its high resolution and sensitivity, can help detect more detailed variations in pulse signals. Photoacoustic imaging^161^ offers a more interpretable approach to observing pulse dynamics.
- Improving evaluation criteria. Considering the characteristics of non-mechanical BP monitoring, a standardized validation protocol should incorporate the following requirements: 1) Reference Devices: Given the trend towards continuous monitoring, it is essential to determine whether a clinical catheter-based device should be used as reference^2^) Sensor-Specific Validation Criteria: Each sensor type requires tailored validation to ensure accuracy, such as the skin tone sensitivity of PPG. 3) Location Requirements: Specify the allowable range of positions for sensor placement, as non-mechanical sensors are sensitive to the physical posture and activity of the subjects. 4) Dataset Separation: To ensure generalization, training and testing datasets should be separated by distinct populations reasonably in deep learning model. 5) Calibration efficiency: Assess its stability over time and across different activities and environmental conditions.
- Investigating the relationship with diseases. Different monitoring technologies may be better suited for specific sub-types of hypertension patients and populations. For instance, contactless systems are particularly advantageous for long-duration nocturnal monitoring, wearable sensors with high sampling rates and motion tolerance are more appropriate for tracking BP dynamics of orthostatic hypertension^162^. In specific populations, such as pregnant women, adolescents, and cardiovascular patients, monitoring priorities vary significantly in terms of required accuracy, frequency, and safety. Additionally, exploring the relationship between BP and CVDs is likely to become a key focus of future research.

## Conclusion

Accurate, continuous, and user-friendly BP monitoring is essential for addressing the global burden of hypertension. As conventional cuff-based methods face limitations in comfort and dynamic tracking, intelligent BP measurement systems have emerged as promising alternatives, driven by advances in sensing and computing technologies. This review aims to serve as a roadmap for researchers, clinicians, and device developers working toward the goal to bridge the gap between technical innovation and clinical application. In this review, we re-frame the landscape of BP monitoring through a new classification based on measurement principles in mechanical and non-mechanical methods. We provide a comprehensive analysis of non-mechanical methods, highlighting innovations across the full pipeline from signal acquisition to calibration. Moreover, we emphasize the critical yet under-explored role of clinical validation. By reviewing current standards and evaluating existing devices, we identify key gaps and call for updated validation frameworks that align with the unique characteristics of next-generation cuffless systems.

## Data Availability

No datasets were generated or analyzed during the current study.
